# Implications of Habitat Loss on Seed Predation and Early Recruitment of a Keystone Palm in Anthropogenic Landscapes in the Brazilian Atlantic Rainforest

**DOI:** 10.1371/journal.pone.0133540

**Published:** 2015-07-17

**Authors:** Leiza Aparecida S. S. Soares, Deborah Faria, Felipe Vélez-Garcia, Emerson M. Vieira, Daniela C. Talora, Eliana Cazetta

**Affiliations:** 1 Programa de Pós-Graduação em Ecologia e Conservação da Biodiversidade, Laboratório de Ecologia Aplicada à Conservação, Universidade Estadual de Santa Cruz, Ilhéus, Bahia, Brazil; 2 Laboratório de Ecologia de Vertebrados, Departamento de Ecologia, Instituto de Ciências Biológicas, Universidade de Brasília, Brasília, DF, Brazil; University of Oxford, UNITED KINGDOM

## Abstract

Habitat loss is the main driver of the loss of global biodiversity. Knowledge on this subject, however, is highly concentrated on species richness and composition patterns, with little discussion on the consequences of habitat loss for ecological interactions. Therefore, a systemic approach is necessary to maximize the success of conservation efforts by providing more realistic information about the effects of anthropogenic disturbances on natural environmental processes. We investigated the implications of habitat loss for the early recruitment of *Euterpe edulis* Martius, a keystone palm in the Brazilian Atlantic Forest, in nine sampling sites located in landscapes with different percentages of forest cover (9%-83%). We conducted a paired experiment using *E*. *Edulis* seeds set up in experimental stations composed of a vertebrate exclosure versus an open treatment. We used ANCOVA models with treatments as factors to assess the influence of habitat loss on the number of germinated seeds, predation by vertebrates and invertebrates, infestation by fungi, and number of seedlings established. Habitat loss did not affect the probability of transition from a dispersed to a germinated seed. However, when seeds were protected from vertebrate removal, seedling recruitment showed a positive relationship with the amount of forest cover. Seed infestation by fungi was not significant, and seed predation was the main factor limiting seed recruitment. The loss of forest cover antagonistically affected the patterns of seed predation by vertebrates and invertebrates; predation by invertebrates was higher in less forested areas, and predation by vertebrates was higher in forested areas. When seeds were exposed to the action of all biotic mortality factors, the number of recruited seedlings was very low and unrelated to habitat loss. This result indicates that the opposite effects of seed predation by vertebrates and invertebrates mask a differential response of *E*. *edulis* recruitment to habitat loss.

## Introduction

Habitat loss is the main driver of the current extinction rates [1–5]. The drastic reduction in tropical forest, where most species are concentrated, poses a serious threat to the maintenance of biological diversity worldwide [6,7]. The remaining forest fragments are often surrounded by anthropogenic landscapes and are generally characterized by few and small remnants scattered within inhospitable matrices that often hamper the movement of certain species [8–10].

The combined effects of habitat loss and fragmentation shape the overall changes in the structure of remaining forest fragments. These small and isolated remnants are further subjected to pronounced edge effects [11], increased light incidence [12,13], and overall changes in species turnover [14,15], leading to profound modifications in biotic interactions, with subsequent consequences for ecological processes and ecosystem functioning [16,17].

Despite the large body of information available on the consequences of habitat loss and fragmentation for biodiversity, current knowledge is highly concentrated on patterns of species richness and abundance, mostly biased toward specific taxonomic groups [3,18–21]. The available information on how ecological processes such as plant-animal interactions [22] and forest functioning [23] are impacted by such drivers is still scarce. Because a reduction in diversity may result in a loss of functional groups [14,24], possibly causing shifts in or disruption of essential ecosystem services [25], the evaluation of ecological processes within this context is essential for maximizing the success of conservation efforts. Thus, a systemic approach can provide more realistic information about the future of natural environments and the impacts of anthropogenic disturbances [26].

Certain ecological processes are of considerable importance to the maintenance of tropical forests, such as those related to plant recruitment [27–29]. Among the limiting factors that govern the establishment of new individuals within communities, those involved in the transition from seed to seedling are relatively unpredictable [29]. This unpredictability stems mainly from the fact that after dispersion, seeds are subject to the effects of myriad biotic and abiotic factors [30]. These factors are highly variable due to the environmental conditions in which the seeds are deposited, and different intensities of seedling mortality are observed as a consequence of such variability [31].

Seed predation is a key biotic factor that limits plant recruitment. This process can significantly reduce the number of viable seeds available [32], which is more crucial than microhabitat differences in determining the survival of certain species [33]. Seeds can be preyed upon by both vertebrates and invertebrates, which may play key roles in limiting recruitment [34,35]. For this reason, understanding how these limiting factors act in areas with different levels of human disturbance is necessary to improve management strategies to increase the likelihood of seedling recruitment [36].

The evaluation of the main factors limiting species recruitment in anthropogenic landscapes becomes particularly important when the players involved in the interactions have key roles in the structure and functioning of forest ecosystems. In this context, the Atlantic Forest endemic palm *Euterpe edulis* Mart. is one of the most important species from the Arecaceae family. This palm can produce a large number of fruits in preserved areas (174.3 kg ha ^-1^yr^-1^; [37]), providing food for at least 58 bird and 21 mammal species [38]. Moreover, this species is exploited for human consumption of the palm heart (apical meristem), the extraction of which leads to the death of the individual [39]. Palm-heart harvesting can result in significant shifts in the regeneration dynamics of the Atlantic Forest by altering the seed-rain density, richness, and composition of functional groups [40]. Thus, understanding how different limiting factors act as demographic bottlenecks in *E*. *edulis* is essential to the future conservation not only of this palm species but also of the animals that consume its fruits and the dynamics of Atlantic Forest as a whole.

In the present study, we evaluated the likelihood of the initial establishment of *E*. *edulis* along a gradient of forest cover at the landscape level. We experimentally assessed the seed germination, post-dispersal predation, and early recruitment of the seeds sampled in nine forest sites located within landscapes ranging 9% to 83% of remaining forest cover. We specifically evaluated (1) seed predation patterns by invertebrates versus vertebrates on *E*. *edulis* seeds; (2) the richness and abundance of small rodents, known as relevant seed predators of this palm [38,41,42]; and (3) the adult density and fruit production of *E*. *edulis* during one year.

We predicted that seed predation by vertebrates would decrease in less forested landscapes as a result of the reduction in small rodent diversity [43]. By contrast, we predicted an increase in seed predation by invertebrates and infestation by fungi in both vertebrate-protected and open treatments in more deforested landscapes, where we expected higher resource availability due to seed dispersal failure or a reduction in predation/removal by vertebrates. Finally, we predicted a higher recruitment of *E*. *edulis* in sites located in more forested landscapes, mainly vertebrate-protected seeds, due to the more suitable abiotic conditions for recruitment in these areas.

## Materials and Methods

### Study site

We conducted the study in anthropogenic landscapes from the Southern Bahia Atlantic Forest. The vegetation is classified as dense ombrophilous forest [44]. The region contains some of the last remnants of the Atlantic Forest in northeastern Brazil [45], harboring some of the largest patches of original forests that are currently protected. For instance, the area includes the Federal Protected Area Una Biological Reserve (Rebio UNA), a conservation unit established in 1980 composed of 18,500 ha, one of the largest blocks of forest in southern Bahia [46]. The landscapes comprise a mosaic of different successional stages, including mature and secondary forests. Otherwise, a substantial area of the forest is actually composed of shade cocoa (*Theobroma cacao*) and rubber plantations [47,48]. *Eucalyptus* plantations are also present, but they are predominant in the southern region of the study area [49].

**Fig 1:**
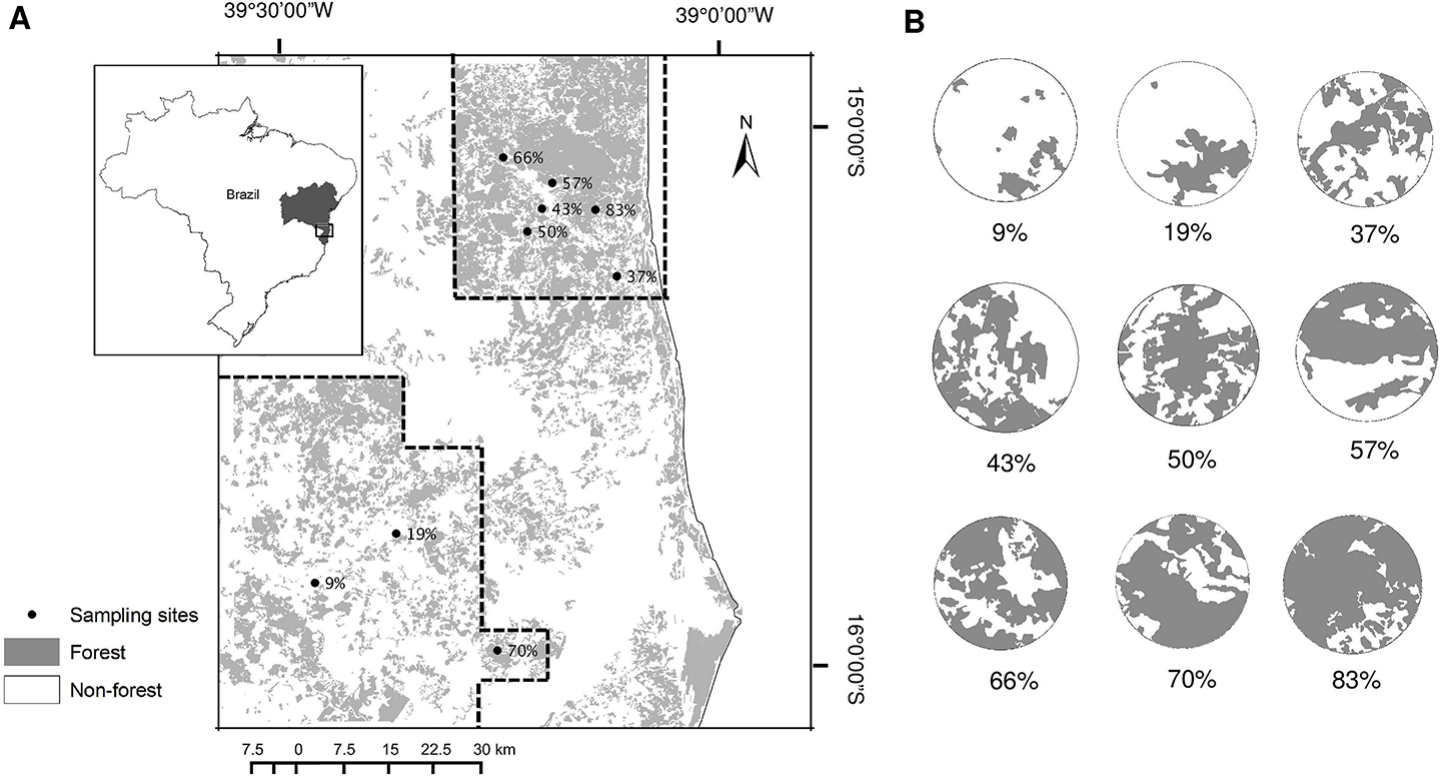
Map depicting the nine study sites. A: Atlantic Forest remnants (gray areas) and the 9 sampling sites (black circles). Dashed lines represent the mapped areas for this study. The images outside the dashed lines were obtained from forest cover map “Atlas dos Remanescentes Florestais da Mata Atlântica” with open access [51]. B: Detail of each site represented by the percentage of forest cover at the landscape scale (~13 km^2^) and comprises a gradient ranging from 9% to 83% located in the Southern Bahia Atlantic Forest.

The present study is part of a research network on the ecological functioning of forest landscapes (REDE SISBIOTA) aimed at evaluating how habitat loss affects regional biodiversity patterns and processes in anthropogenic landscapes. The sampling design of the REDE SISBIOTA was built by mapping the southern Bahia region with the aid of satellite images specifically acquired (QuickBird and WorldView, from 2011) and already available (RapidEye, from 2009–2010). After an intensive ground-truthing, we elaborated a map of the land use of 3,470 km^2^, including the municipalities of Una, Belmonte, Canavieiras, Santa Luzia, and Mascote (15° 28’S, 39° 15’W; coordinates obtained in the center of the sample area). Our map was used to visually identify and quantify the different forest categories at a scale of 1:10,000. For the present study, however, we considered only the amount of native vegetation in the landscape in our forest cover estimate. All crops, including shaded cacao plantations, were excluded from our quantification. Based on this map, we identified 48 forest sites with a minimum distance of 1 km from each other, all located within the limits of native forest fragments. Using ArcGIS (9.3), we delimited an area with a 2 km radius from the center of each forest fragment and estimated the total forest cover in each of these ~13 km^2^ landscapes. This sample design is called patch-landscape scale, and this methodology is advantageous because it allows the association of site-scale response variables with landscape-scale attributes [50]. For the present study, we randomly selected nine forest sites, herein referred to as sampling sites, covering a wide variation from 9% to 83% of forest cover at the landscape scale to evaluate the seed predation and early recruitment of *E*. *edulis* (Fig 1). The nine sample sites were located at least 2km from each other, to guarantee no overlap and provide independence among landscapes.

### Recruitment experiment

We evaluated seedling recruitment by monitoring the fate of *E*. *edulis* seeds. In each forest fragment, we established a 0.5 ha (100 m by 50 m) plot and randomly conducted a paired experiment in fifteen stations. Each station was composed of an open and a closed treatment arranged side by side. In the closed treatment, we placed five *E*. *edulis* seeds inside a 0.04 m^2^ cage that allowed only the passage of light and insects but excluded vertebrate access. In the open treatment, five seeds were set up together on the forest floor without the protection of the cage, thus enabling access by all animal groups. We placed 150 seeds in each site in both treatments (open and closed), totaling1,350 seeds. The seeds used in this experiment were taken from the mature fruits of five adults from two different forest fragments without plot installation. We removed the pulp from all seeds (epicarp and mesocarp), simulating the action of animal dispersers [52]; mixed the seeds of all adults; and randomly selected seeds for use in the experiment. We started the experiment in June 2013, the peak month of *E*. *edulis* fruit production in our sites. The experimental stations were observed once per month for a period of six months, a sufficient time lag for observing the early establishment of seedlings. During each visit, we recorded the seed state, which was categorized into six distinct categories: (1) *germinated*—with radicle emission of approximately 1 mm, including seeds that had signs of invertebrate attack but were germinating; (2) *invertebrate predation*—when seeds exhibited a typical entrance hole left by beetles [41,53]; (3) *vertebrate predation*—when the seed was carried to an area outside the experiment area or when it presented typical teeth marks caused by rodents [53]. We considered the seeds removed to have been predated by vertebrates because this assumption has been shown to be largely valid [54], and a recent study showed that secondary dispersal of *E*. *edulis* seeds is negligible [55]; (4) *fungi infested*—with signs of pathogen attack; (5) *intact*—with no signs of previous attack; (6) *seedling*—individuals with an open green leaf, palmate leaves, and still presenting the seed with endosperm reserves [56] (S1 Fig).

Germinated seeds (state 1) were just evaluated in the first month of the experiment, and all other states were evaluated monthly for six months. For state 1, we summed the data of germinated seeds and seeds predated by invertebrates that also germinated because we were mainly interested in the germination process and in factors limiting seed germination. Moreover, we evaluated seed predation by invertebrates and all seeds classified as state 1 in the first month that were also predated by invertebrates, did not recruit after six months. The seed state classification was based on the first fate, therefore monthly inspections were necessary (i.e. seeds predated by invertebrates that were attacked by fungus later, were categorized as predated by invertebrates). We used the total seed number into the same category to calculate the percentage of each state from the overall seeds available in the beginning of the experiment.

### Small mammal survey

A large number of mammals are known to act as *E*. *edulis* seed predators [41,42,53,55,57–59]. In our studied landscapes, however, large mammals such as tapirs (*Tapirus terrestris)* and white-lipped peccaries (*Tayassu pecari)* are locally extinct and medium-sized mammals such as pacas (*Cuniculus paca)* and agoutis (*Dasyprocta* spp.) still occur but in very low densities [60]. For this reason, we assessed only the assemblage of small non-volant mammals [42] and subsequently classified those species as seed predators based on previous literature or on the authors’ knowledge.

We sampled the small mammals using live traps (30x9x8 cm Sherman type and 45x16x16 cm Tomahawk type) placed both on the ground and on tree branches or vines. In each site, we established three pairs of 100 m parallel trap-lines spaced 300 m apart, with 50 m between lines in the same pair. Each line contained six equidistant stations in which we set up two traps (one of each type) on the ground and in trees (1–2 m above the ground). We baited the traps with a mixture of banana, corn meal, peanut butter, and cod liver oil and checked them daily for ten capture days per plot. In each site, we conducted one trapping survey, totaling 720 trap-nights per site and 6,480 trap-nights in total. Captured individuals were identified, marked with ear tags, and released immediately after these procedures.

### *Euterpe edulis* density and fruit production

In each 0.5 ha plot, we surveyed all *E*. *edulis* adults. Only individuals with DBH ≥ 5 cm with past or present reproductive signs were considered adults. The individuals were marked and monitored for one year for the evaluation of fruit production. We conducted monthly phenological observations to evaluate the presence of mature fruits and counted the number of infructescences occurring in each individual.

### Ethics statement

The study was conducted in private areas and all landowners gave permission to sample in their properties. The mammal capture procedures were approved by the Instituto Brasileiro de Meio Ambiente e dos Recursos Naturais–IBAMA (license number 38515–2) and followed the guidelines of the American Society of Mammalogists for the use of wild mammals in research [61]. Our study involved only the capture, identification, ear tag marking, and the immediate release of small rodents. No invasive procedures were performed and no animals were sacrificed during this study. We received an approval from the Committee for Animal Use (Comitê de ética no uso de Animais CEUA-UESC, http://www.uesc.br/ceua/) from the Universidade Estadual de Santa Cruz (Process number 003/2013). The mammals surveyed are not endangered or protected species.

### Data analysis

We analyzed the relation between the seed state along the gradient of forest cover with analyses of covariance (ANCOVA), in which we considered the mean percentage of the number of seeds in each state as response variables, the treatment (open and closed) as a factor, and the percentage of forest cover in each landscape as a covariate. Therefore the total sample size was 18 (9 forest sites with two treatments).To perform the ANCOVA, we previously tested data normality by applying the Shapiro-Wilk test and variance homoscedasticity by applying the Bartlett test. We also evaluated the homogeneity of slopes by adding the interaction term in the ANCOVA. When slopes were not homogenous, we performed linear regressions for each treatment. Prior to the analyses, all percentage values were arcsine-square-root transformed.

To evaluate vertebrate seed removal, we performed a simple linear regression, considering the number of seeds removed from the open treatment as the response variable, as vertebrates had no access to the closed treatment and no seeds were observed being removed from the cages.

We also performed linear regressions to evaluate how forest cover influences small rodent richness and abundance and how these factors influence seed predation by vertebrates. We assessed whether palm density, the percentage of fruiting individuals, and the number of infructescences influence the total number of seeds predated by vertebrates and invertebrates (considering both open and closed treatments). All linear regressions used the percentage of forest cover in the nine sample sites as the independent variable. The statistical analyses were performed using the R software environment, version 2.15.0 (R Development Core Team 2012).

## Results

### Recruitment experiment

**Fig 2:**
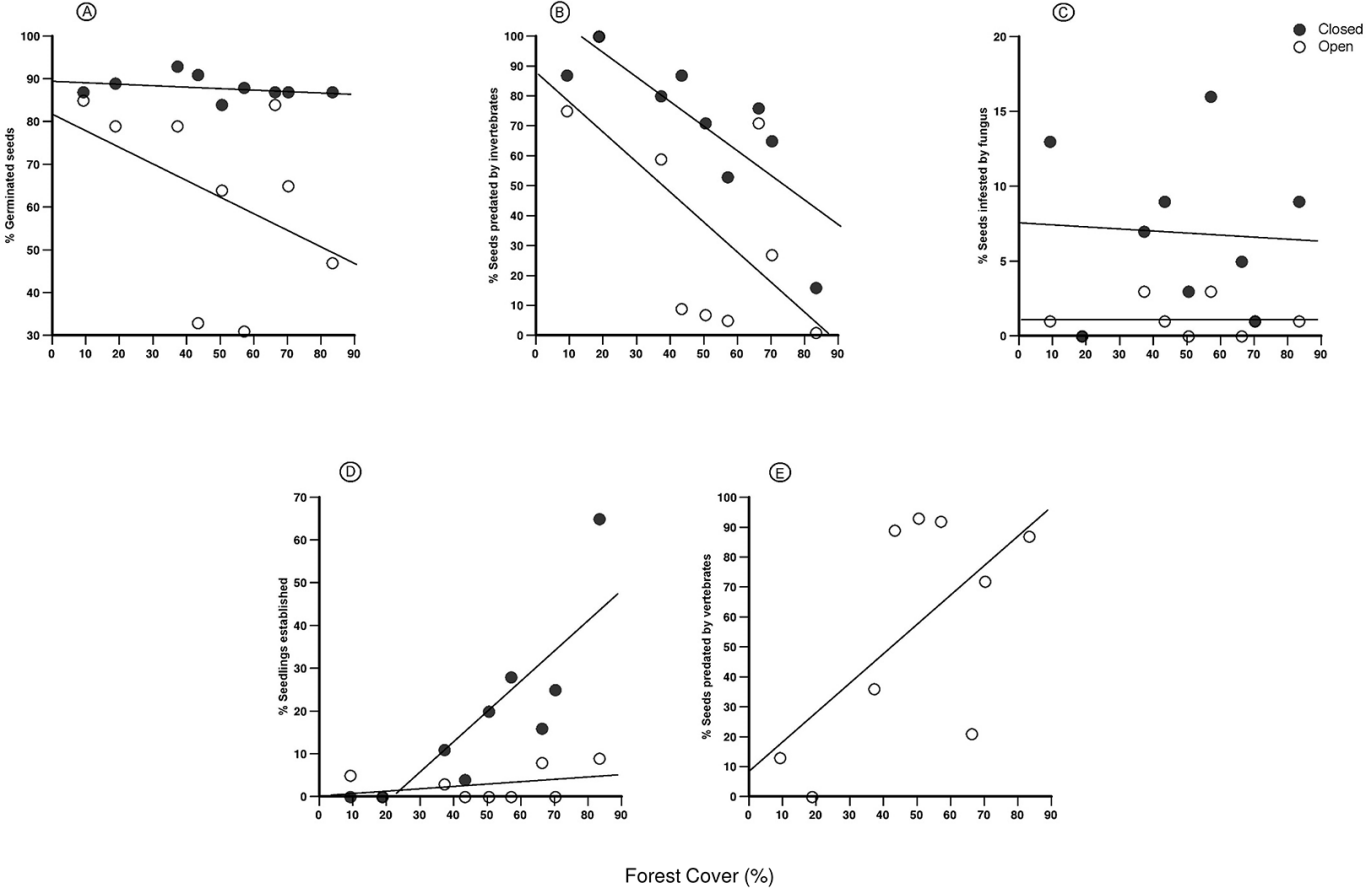
Mean percentage of *E*. *edulis* seeds in each state. A. germinated seeds; B. seeds predated by invertebrates; C. seeds infested by fungus; D. recruited seedlings; E. seeds predated by vertebrates along a gradient of forest cover in the Southern Bahia. In the legend “open” and “closed” refer to the experimental treatments.

Of the 1,350 seeds available when the experiment started, 1,019 germinated (75.5%) during the first month. The percentage of germinated seeds was significantly higher for the closed treatment, with no significant influence of forest cover reduction in the landscape (Table 1 and Fig 2). Differences between treatments were most likely due to the high rates of seed removal in the open treatment, mainly observed in three sites with intermediate to high levels of forest cover (43%, 57%, and 83% of forest cover). In these sites, we found that 48.0%, 62.7%, and 60.0% of seeds were removed, respectively. As a result, total seed germination was reduced from 88.0% to 62.9% for the closed treatment versus the open treatment, respectively (S1 Table).

**Table 1:** Results of the univariate ANCOVA.

	df	MS	*F*	*P*
**Germination**				
Treatment	1	0.387	15.701	0.001
Forest cover (covariate)	1	0.047	1.945	0.185
Treatment x Forest cover	1	0.029	1.2	0.292
Error	14	0.024		
**Infested by fungus**				
Treatment	1	0.110	8.968	0.010
Forest cover (covariate)	1	0.001	0.046	0.833
Treatment x Forest cover	1	0.001	0.004	0.949
Error	14	0.012		
**Invertebrate Predation**				
Treatment	1	0.627	6.471	0.023
Forest cover (covariate)	1	135.331	13.961	0.002
Treatment x Forest cover	1	0.020	0.208	0.655
Error	14	0.097		

Effects of treatment (two levels: vertebrate exclosure versus open treatment) on the mean percentage of germinated seeds, seeds infested by fungus, and invertebrate predation along the gradient of forest cover reduction in southern Bahia, Brazil.

Six months after the beginning of the experiment, of the total of 1,350 seeds available, 738 (54.7%) had been predated by invertebrates, including 473 (64.09%) in the closed treatment and 265 (35.91%) in the open treatment. Although we found a significant difference between the open and closed treatments, forest cover reduction at the landscape scale positively influenced seed predation by invertebrates in both treatments (Table 1 and Fig 2). Additionally, seed predation by vertebrates was responsible for the loss of 373 seeds, which represented 55.26% of the total number of available seeds in the open treatment. Forest cover reduction tended to negatively affect seed predation by vertebrates, which was the opposite pattern observed for seed predation by invertebrates. Despite a clear declining trend toward areas with a low percentage of forest cover, the response of the removal rates of seeds was not significant (Linear Regression, r^2^ = 0.31, *p* = 0.07).

Seed infestation by fungi was a minor factor influencing seed mortality in our experiment, responsible for the damage of only 57 seeds (4.2%). Although the mean percentage of seeds infested by fungi differed between the open and closed treatments, it was not affected by forest cover reduction in the landscape (Table 1 and Fig 2).

We found a marked reduction in the number of seeds that successfully reached the stage of established seedlings. Of 1,019 palm seeds that germinated in the first month (summed open and closed treatments), 145 (14.2%) turned into seedlings at the end of the sixth month. Of these seedlings, 126 recruited in the closed treatment and only 19 seedlings recruited in the open treatment. We did not perform the ANCOVA model to evaluate the recruitment because the slopes were not homogenous (treatment x forest cover, F_1,14_ = 13.70, *p* = 0.002). The linear regressions indicated that forest cover reduction in the landscape did not affect the early recruitment of seedlings in the open treatment (r^2^ = -0.102, *p* = 0.627), whereas it strongly affected the recruitment in the closed treatment (r^2^ = 0.832, *p*< 0.0001) (Fig 2).

### Small mammal survey

We sampled a total of 71individuals belonging to six species of rodents that were potential predators of *E*. *edulis* seeds (S2 Table). Linear regression results showed that forest cover reduction negatively influenced rodent species richness (r^2^ = 0.62, *p*< 0.01) but did not influence abundance (r^2^ = -0.13, *p* = 0.79). Additionally, the mean percentage of seeds preyed upon by vertebrates was related to species richness (r^2^ = 0.41, *p* = 0.04) but not directly related to small rodent abundance (r^2^ = 0.07, *p* = 0.25).

### *E*. *edulis* density and fruit production

The results from linear regressions indicated that the mean percentage of seeds preyed upon by vertebrates was not related to palm density (r^2^ = 0.19, *p* = 0.14), the number of fruiting individuals (r^2^ = 0.18, *p* = 0.14), or the number of mature infructescences produced (r^2^ = 0.13, *p* = 0.18) in each site. Similarly, the mean percentage of seeds predated by invertebrates (closed + open treatment) was not related to palm density (r^2^ = 0.36, *p* = 0.051), the number of fruiting individuals (r^2^ = 0.33, *p* = 0.06), or the number of infructescences produced (r^2^ = 0.31, *p* = 0.07) at each site.

## Discussion

We experimentally demonstrated the main factors limiting the early establishment of a keystone palm in Atlantic Forest landscapes and how they were influenced by forest cover reduction at a landscape scale. Our results indicate that seed recruitment is not directly linked to forest cover reduction; however, the main factors that drive this process are antagonistically affected by forest loss. In this context, the effects on the recruitment process might have wider consequences that are not easily detected due to the high complexity of species interaction and the synergetic effects that act on deforested landscapes.

All evaluated landscapes showed high germination rates during the first month of the experiment. This result is uncommon because several studies have reported that defleshed palm seeds usually germinate only after two or three months [38,62–64]. Seed germination might be influenced by a number of factors, such as temperature, pH, and soil humidity [65–67]. However, we can infer that the uniformity and high germination rates found in our study may have reduced seed vulnerability to the possible environmental differences presented in each landscape. Additionally, our experiment revealed that regardless of the amount of forest cover in the landscape, no demographic bottleneck in the first transition stage from dispersed seed to germination was observed.

Despite the high rates of seed germination, at the end of the sixth month, more than 82% of the available seeds had been predated. Seed predation has been considered one of the main mortality factors limiting plant recruitment in several plant studies, which suggests that predation is more critical to seedling recruitment than favorable microsite limitations, pathogenic attack or herbivory [68–71]. Higher rates of *E*. *edulis* seed predation (99.7%) were also found on Anchieta Island in Brazil after 30 days of seed exposition time due to the extremely high densities of agoutis in that area [59]. However, the differences in methodologies, primarily in the exposition time, used in seed predation studies make comparative analyses difficult. The few studies that used the same or higher exposition times as ours and that provided raw predation results reported much lower predation rates (ranging from 7% to 23% of the seeds) [52]. For this reason, we reiterate that seed predation of *E*. *edulis* was the main limiting factor to the early recruitment of this species in our landscapes.

The high percentage of seed predation in all forest fragments was the result of the combined action of both vertebrates and invertebrates. However, the behaviors of these two groups followed opposite trends along the gradient of forest cover. Invertebrate seed predation was higher in less forested landscapes, whereas vertebrate seed predation was higher in more forested landscapes. Although the relation between vertebrate seed predation and the degree of forest cover was not statistically significant, there was a clear declining trend toward less forested areas. This finding is also corroborated by the increase in recruitment found only in the closed treatment, where vertebrates had no access to the seeds, and by the observed increase in rodent species richness along the forest-cover gradient. A similar opposing pattern among vertebrate and invertebrate seed predation was found for the palms *Astrocaryum aculeatissimum* [72] and *E*. *edulis* [55] in Atlantic Forest fragments.

Additionally, we did not find any relationship between the number of seeds predated by vertebrates and invertebrates and the number of palm individuals or palm fruit availability. Palm seed predation by invertebrates is usually performed by specialist insects that consume one or few taxonomically related groups [73,74]. For these invertebrates, an increase in resource availability generally is positively associated with predator abundance [34,41]. Our results indicated, however, that resource availability is not the mechanism that drives seed predation patterns, especially concerning invertebrate activity.

The influence of forest cover reduction on the patterns of vertebrate and invertebrate seed predation corroborated our initial hypothesis. Considering vertebrate predation, however, we observed a significant effect of forest cover only on rodent richness and not on the overall abundance of seed-eaters. An increase in forest cover results in an increase in complexity that may explain the observed increase in small rodent diversity [75]. The lack of a relation between forest cover and abundance that we observed might have been caused by either biological (*e*.*g*., territoriality, non-linear responses to deforestation) or methodological factors (*e*.*g*., differences in trapability among species). The evaluation of such factors is beyond the scope of the present study. We highlight, however, that of six seed-eating species registered in this study, five were found only in areas with above 40% forest cover, which reinforces the value of these landscapes for sustaining small-rodent diversity. A similar result was observed by [43], who found a rapid decline in small mammal abundance and richness in landscapes with less than 30% forest cover.

The increasing invertebrate seed predation in less forested areas indicates that the absence of other seed predators or secondary seed dispersers, such as pacas and agoutis, might reduce resource competition, and as a consequence, the seeds were available for longer periods. A similar situation was also found by [34], who reported that the seeds of *Attalea dubia* were highly predated by invertebrates in the absence of *Sciurus ingrami*, the main seed predator and secondary disperser of this species. Additionally, the increase in seed predation by invertebrates was expected in less forested areas due to failures in seed dispersal [72,76]. For instance, [77] and [78] showed that forest cover has a stronger influence than individual patch size on the distribution of frugivorous birds in rainforest fragments, a result with possible consequences for seed dispersal in deforested areas. Therefore, the failure in seed dispersal in those areas might result in seed aggregation below the mother plant [72,79], and such high seed density can promote the attraction of seed-predator insects [80].

The third mortality factor evaluated, infestation by fungi, was not influenced by forest cover reduction and was unrepresentative in all evaluated landscapes. The low number of infested seeds was previously expected in our experiment because we used defleshed seeds. *E*. *edulis* pulp is sugar rich [81], which is the main factor responsible for rapid microorganism growth that triggers embryo loss [67]. The mean number of seeds infested by fungi was higher in the vertebrate exclosure than in the open treatment, a similar pattern as that found for seed predation by invertebrates. The differences between treatments were not expected but can be explained by the higher rates of seed removal in the open treatment. Therefore, the number of seeds available to fungi or invertebrates sharply decreased in the open treatment after the first month.

In contrast to seed germination, the transition from germinated seed to seedling was a critical step to the early recruitment of *E*. *edulis* seeds. Seedling recruitment was low in all sample sites, but we found a distinct pattern in seedling establishment between treatments. Forest cover reduction strongly influenced the percentage of establishment in the closed treatment but not in the open treatment, which suggests that independently of the amount of forest cover in the landscape, dispersed seeds of *E*. *edulis* demonstrated low establishment capacities. This low recruitment rate was also observed in different studies in which the seeds were subject to high seed predation activities [36,59,82,83]. However, we suggest that the selective pressures exerted in the less forested areas are likely more harmful to long-term species maintenance. The cumulative effects of high mortality rates, adverse microclimatic conditions, and increased susceptibility to harvest might hamper the persistence of this keystone species in these areas.

In conclusion, we have shown empirically how habitat loss impacts biodiversity, more specifically the negative effects of changes in species interactions to plant recruitment. The consequence of losses in species interaction for biodiversity was recently proposed as an emerging and priority field [84]. We also showed that distinct agents (invertebrate versus vertebrate) present contrasting predation responses along a gradient of forest cover, but as a consequence of multiple species interaction, the early recruitment of *E*. *edulis* is low in all sampled landscapes in southern Bahia. Because declines in recruitment are presumed to underlie plant extinctions in altered habitats [85–87], we suggest seed sowing and seed protection in forested areas as possible management strategies for ensuring the maintenance of *E*. *edulis* populations over time.

## Supporting Information

S1 Fig Pictures illustrating the six states of seeds. A. germinated; B. predated by invertebrate; C.: predated by vertebrate; D. infested by fungi; E. intact; F. seedling.
(TIFF)

S1 Table Number of seeds in each treatment. Number of germinated seeds, seeds predated by invertebrates and vertebrates, seeds infested by fungus and recruited seedlings in the open and closed treatments along the gradient of forest cover reduction in southern Bahia, Brazil.
(DOCX)

S2 Table Abundance of rodent potential predators of *E*. *edulis* seeds. Number of small rodents found along the gradient of forest cover reduction in southern Bahia, Brazil.
(DOCX)
