# Supplementary figures and images for: Implications of Habitat Loss on Seed Predation and Early Recruitment of a Keystone Palm in Anthropogenic Landscapes in the Brazilian Atlantic Rainforest
Source: PLoS One. 2015 Jul 17;10(7):e0133540. doi: 10.1371/journal.pone.0133540 (PMC4505908; doi:10.1371/journal.pone.0133540)

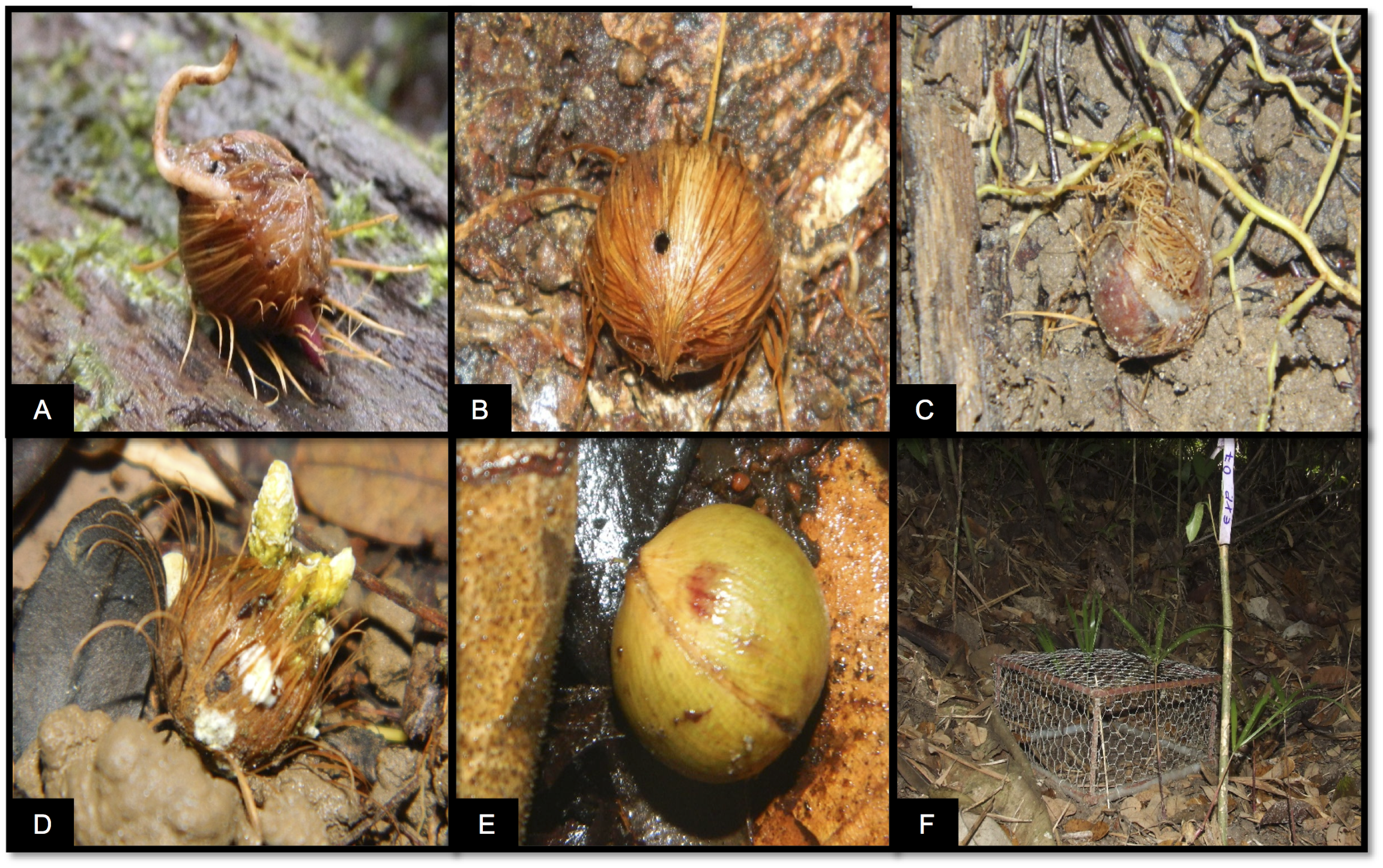

Supplement: S1 Fig — A. germinated; B. predated by invertebrate; C.: predated by vertebrate; D. infested by fungi; E. intact; F. seedling. (TIFF) [file pone.0133540.s001.tiff]
