# Supplementary material for: Implications of Habitat Loss on Seed Predation and Early Recruitment of a Keystone Palm in Anthropogenic Landscapes in the Brazilian Atlantic Rainforest
Source: PLoS One. 2015 Jul 17;10(7):e0133540. doi: 10.1371/journal.pone.0133540 (PMC4505908; doi:10.1371/journal.pone.0133540)
Supplement: S1 Table — Number of germinated seeds, seeds predated by invertebrates and vertebrates, seeds infested by fungus and recruited seedlings in the open and closed treatments along the gradient of forest cover reduction in southern Bahia, Brazil. (DOCX) [file pone.0133540.s002.docx]

**S1 Table. Number of seeds in each treatment.** Number of germinated seeds, seeds predated by invertebrates and vertebrates, seeds infested by fungus and recruited seedlings in the open and closed treatments along the gradient of forest cover reduction in southern Bahia, Brazil.

| Forest Cover (%) | Closed Treatment | | | | Open Treatment | | | | |
| --- | --- | --- | --- | --- | --- | --- | --- | --- | --- |
|  | Fungus | Invertebrate | Germinated | Seedling | Fungus | Invertebrate | Germinated | Seedling | Vertebrate |
| 9 | 10 | 65 | 65 | 0 | 1 | 56 | 64 | 4 | 10 |
| 19 | 0 | 75 | 67 | 0 | 0 | 75 | 59 | 0 | 0 |
| 37 | 5 | 60 | 70 | 8 | 2 | 44 | 59 | 2 | 27 |
| 43 | 12 | 40 | 66 | 21 | 2 | 4 | 23 | 0 | 69 |
| 50 | 7 | 65 | 68 | 3 | 1 | 7 | 25 | 0 | 67 |
| 57 | 2 | 50 | 63 | 14 | 0 | 5 | 48 | 0 | 65 |
| 66 | 4 | 57 | 65 | 12 | 0 | 53 | 63 | 6 | 16 |
| 70 | 1 | 49 | 65 | 19 | 1 | 20 | 49 | 0 | 54 |
| 83 | 7 | 12 | 65 | 49 | 1 | 1 | 35 | 7 | 65 |
