# Supplementary material for: Implications of Habitat Loss on Seed Predation and Early Recruitment of a Keystone Palm in Anthropogenic Landscapes in the Brazilian Atlantic Rainforest
Source: PLoS One. 2015 Jul 17;10(7):e0133540. doi: 10.1371/journal.pone.0133540 (PMC4505908; doi:10.1371/journal.pone.0133540)
Supplement: S2 Table — Number of small rodents found along the gradient of forest cover reduction in southern Bahia, Brazil. (DOCX) [file pone.0133540.s003.docx]

**S2 Table**. **Abundance of rodent potential predators of *E*. *edulis* seeds**. Number of small rodents found along the gradient of forest cover reduction in southern Bahia, Brazil.

| Species of small rodents | Forest cover (%) | | | | | | | | |
| --- | --- | --- | --- | --- | --- | --- | --- | --- | --- |
|  | 9 | 19 | 37 | 43 | 50 | 57 | 66 | 70 | 83 |
| *Akodon cursor* | - | - | - | - | - | - | - | 2 | 2 |
| *Cerradomys* sp. | - | - | - | - | - | - | - | 3 | - |
| *Euryoryzomys russatus* | - | - | - | 2 | - | - | - | - | - |
| *Hylaeamys laticeps* | - | 1 | 16 | 22 | 2 | 9 | 1 | - | 1 |
| *Nectomys squamipes* | - | - | - | - | 1 | - | - | - | 1 |
| *Oligoryzomys nigripes* | - | - | - | 1 | - | 1 | 4 | - | 2 |
